# Dr. Thomas Jackson

**Published:** 1900-02

**Authors:** 


Dr. Thomas Jackson, U. of B., '71, of Whitehaven, Eng., died at his residence in that city, December 4, 1899, aged 65 years. Soon after graduation he returned to his native place and resumed the practice of medicine. He was at the time of his death the senior staff surgeon of the Whitehaven and West Cumberland Infirmary, manager of the Whitehaven Savings Bank, President of the Whitehaven Choral Union, and member of the Public Library and Technical Education Committee.

At the monthly committee meeting of the Whitehaven and West Cumberland Infirmary, held the day after his death, the following resolution was proposed by Mr. W. H. Kitchin (the chairman), seconded by Mr. J. Gunson, and supported by Dr. Muriel, Dr. Muir, and Dr. Welby I'Anson: "That the committee of the Whitehaven and West Cumberland Infirmary places on record its profound regret at the loss sustained by the institution through the death of the late Thomas Jackson, M. D., who for twenty-three years held the appointment of visiting surgeon, and who by his high moral principle and constant devotion to the duties of his office earned the sincere respect of all who were associated with him. The committee also desires to express its deep sympathy with Mrs. Jackson and her family in their great bereavement."

Dr. Jackson was uncle to Dr. William J. Falkner, of Youngstown, N. Y.

